# A Review of the Evidence that Ochratoxin A Is an Nrf2 Inhibitor: Implications for Nephrotoxicity and Renal Carcinogenicity

**DOI:** 10.3390/toxins6010371

**Published:** 2014-01-20

**Authors:** Alice Limonciel, Paul Jennings

**Affiliations:** Division of Physiology, Department of Physiology and Medical Physics, Innsbruck Medical University, Innsbruck A6020, Austria; E-Mail: Alice.Limonciel@i-med.ac.at

**Keywords:** proximal tubule, ochratoxin, oxidative, Nrf2

## Abstract

Several studies have demonstrated that ochratoxin A (OTA) inhibits the nuclear factor, erythroid 2-like 2 (Nrf2) oxidative stress response pathway. At the cellular level this would attenuate (i) glutathione synthesis; (ii) recycling of oxidised glutathione; (iii) activity of oxidoreductases; and (iv) phase II metabolism inducibility. The effects combined would render the cell and tissue more vulnerable to oxidative stress. Indeed, Nrf2 knock out animals exhibit increased susceptibility to various types of chemical-induced injury. Several studies have shown that OTA exposure can inhibit Nrf2 responses. Such an action would initially lead to increased susceptibility to both physiological and chemical-induced cell stress. However, chronic exposure to OTA may also act as a selective pressure for somatic mutations in Nrf2 or its inhibitor Keap-1, leading to constitutive Nrf2 activation. Nrf2 overexpression confers a survival advantage and is often associated with cancer cell survival. Here we review the evidence for OTA’s role as an Nrf2 inhibitor and discuss the implications of this mechanism in nephrotoxicity and carcinogenicity.

## 1. Introduction

The ochratoxin family of mycotoxins is found in a wide variety of foods, including cereals, meat, dried fruits, nuts, coffee, wine and beer. Ochratoxin A (OTA) is considered the most toxic of the family and is found at highest concentrations in European food stuffs. The average daily intake of a European adult is estimated at 1 ng.kg^−1^ b.w., although exposure rates can be up to eight times higher [1,2]. OTA’s main target is the renal proximal tubule where it is taken up from the interstitium via the organic anion transporters (OAT) 1 and 3 and from the lumen via OAT4 [3,4]. While it is also taken up by other tissues, such as the liver, OTA accumulates at higher rates in proximal tubule cells, which is responsible for its renal specificity. OTA is both a nephrotoxin and a renal carcinogen [5]. It has also been shown to cause renal tumors in rats with high metastatic potential [6,7]. Several mechanisms have been proposed for these adverse effects of OTA, including genotoxicity [8], cytoskeleton destabilization [5,9] and histone acetyltransferase (HAT) inhibition [10]. In addition, there is increasing evidence that shows that OTA inhibits the Nrf2 oxidative stress response.

## 2. The Nrf2 Pathway

The nuclear factor, erythroid 2-like 2 (NFE2L2 or Nrf2) constitutes the main oxidative stress response and drives the transcription of genes involved in glutathione synthesis and recycling, phase II metabolism and the reduction of oxygen species and quinones [11]. Under basal conditions, the Nrf2 protein is sequestered in the cytosol by the actin bound Keap-1 homodimer and targeted for ubiquitination by CUL3, an E3 ubiquitin ligase [11]. Keap-1 contains specific cysteine thiol residues that are sensitive to oxidative and electrophilic stress and, once modified, inhibit Keap-1’s ability to bind Nrf2. Unbound Nrf2, a member of the cap “n” collar (CNC) subfamily of bZIP transcription factors, translocates to the nucleus, heterodimerises with members of the small maf protein family and initiates transcription by binding to antioxidant/electrophilic response elements (ARE/EpRE) [12,13]. The ARE/EpRE is contained within the promoters of several genes including those involved in (i) *glutathione synthesis and recycling*, comprised of glutamate-cysteine ligase, catalytic and modifier subunits (GCLC, GCLM), glutathione reductase (GSR); (ii) *genes with antioxidant properties*, such as heme oxygenase 1 (HMOX1), NAD(P)H dehydrogenase, quinone 1 (NQO1), superoxide dismutase 1 (SOD1), thioredoxin reductase 1 (TXNRD1), sulfiredoxin (SRXN1) and aldo-keto reductases (AKRs); and (iii) *genes involved in xenobiotic metabolism and transport*, comprised of glutathione-S-transferases (GSTs), UDP-glucuronosyltransferase 1A1 (UGT1A1) and ATP-binding cassette, sub-family C3 (ABCC3) [14,15,16,17,18,19]. Since the activation of Nrf2 results in changes in transcription, mRNA profile of the target genes is an ideal method to detect the complete activation of the pathway.

The Nrf2 response has been shown to be robustly induced in several tissues in response to chemical and physiological stressors. For example, many nephrotoxins induce Nrf2 nuclear translocation and Nrf2 dependent gene induction in renal epithelial cells, including potassium bromate, cadmium chloride, diquat dibromide and cyclosporine A [20,21,22]. This pathway has also been shown to be activated in pulmonary models exposed to heavy metals [23,24]. Additionally, we have recently shown that glucose depletion and subsequent re-introduction can result in Nrf2 activation [25]. Thus activation of the Nrf2 pathway provides a robust transcriptional response to redress an increase in oxidative equivalents, protecting the cell from oxidative stress. This is best illustrated in Nrf2 knock-out mice, which are more susceptible to chemically induced toxicity [26,27,28]. For example Nrf2^-/-^ homozygote mice are far more sensitive to acetaminophen induced hepatotoxicity than wild type or heterozygotes [29].

## 3. OTA: An Inhibitor of Nrf2?

A number of studies have shown that OTA induces oxidative stress and that pre-incubation of antioxidants can protect against OTA induced injury (reviewed in [30]). For example it has been demonstrated that OTA induces a dose-dependent increase in reactive oxygen species (ROS) in primary rat proximal tubule cells and in the LLC-PK1 cell line, leading to a depletion of intracellular glutathione and cell death [31]. Pre-incubation with the anti-oxidant *N*-acetylcysteine (NAC) attenuated these effects [31]. However, no study has reported an Nrf2 activation in response to OTA and a large genome-wide transcriptomic study reported there was no induction of Nrf2 dependent genes by OTA in the five different *in vitro* proximal tubular models tested [5]. This is a rather strange phenomenon for a compound that induces oxidative stress. Thus it is possible that OTA exposure somehow prevents Nrf2 activation. There are several lines of evidence to support this hypothesis.

**Figure 1:**
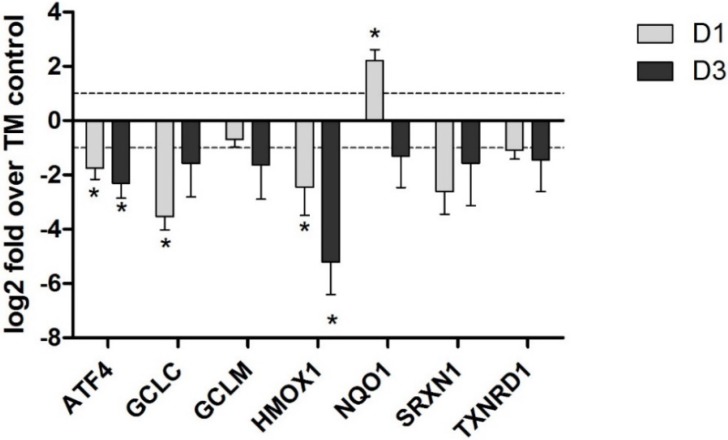
Ochratoxin A (OTA) (5 µM) induced an inhibition of Nrf2-dependent genes in human primary proximal tubule cells. Human primary proximal tubular cells from three different donors were cultured to confluence and treated with 5 µM OTA for 1 (grey bar) and 3 days (black bar). RNA was isolated and ran on Affymetrix HGU-133 plus 2 arrays. Nrf2-dependent genes were selected for graphical representation and are expressed as log 2 fold over time-matched (TM) control ± SEM. See [5] for further details.

Rats exposed to OTA by oral gavage for up to one year exhibited a decrease in the mRNA of Nrf2-dependent genes in the kidneys, but not the liver [32]. This presumably reflects the fact that uptake is higher in the kidney and provides direct evidence for OTA acting as an Nrf2 inhibitor. The genes affected included GCLC, GCLM, glutathione synthetase (GSS), UGT3B5 and multiple GST isoforms. Decreases in protein levels were confirmed for GSTP and GCLC by western blot after 21 days and 12 months of exposure to OTA [32]. In an *in vitro* study we have shown that OTA exposure resulted in an inhibition of Nrf2-dependent genes in human primary proximal tubule cells (Figure 1). Cavin *et al.* [33] have also demonstrated an OTA-induced Nrf2 inhibition by investigating the protein expression of several Nrf2-regulated genes in rat liver and kidney cell cultures. They showed that OTA depleted both renal and hepatic cells of GSH and decreased the protein levels of Nrf2 targets NQO1, GCLC, GSTA5, GSTP1 and AKR7A1.

## 4. Potential Mechanisms of OTA-Induced Nrf2 Inhibition

There are several potential mechanisms for OTA-induced Nrf2 inhibition: (i) inhibition of Nrf2 nuclear translocation; (ii) inhibition of Nrf2 DNA binding; or (iii) epigenetic effects preventing normal Nrf2-dependent transcription.

Independent studies have shown that OTA inhibits Nrf2 activation prior to nuclear translocation [34,35]. Kumar *et al*. exposed mice to various concentrations of OTA topically for 24 h [34]. They demonstrated a dose-dependent increase in ROS and a concomitant decrease in GSH in skin cells. At the highest concentration, a significant increase in DNA damage was observed. However, there was no nuclear accumulation of Nrf2 at any concentration and even a slight decrease in basal nuclear Nrf2 localization was observed [34]. Ramyaa *et al.* exposed cultured kidney cells to OTA and also demonstrated a decrease in Nrf2 nuclear translocation compared to control cells [35]. Furthermore they showed that activating Nrf2 by pre-incubation with the flavonoid, quercetin, prevented OTA-induced cell death [35]. In LLC-PK1 cells, Boesch-Saadatmandi *et al.* demonstrated that co-administration of OTA severely attenuated sulforaphane-induced Nrf2 nuclear translocation and transactivation [36].

It has also been suggested that OTA can interfere with Nrf2 DNA binding. Two independent studies have shown that OTA induces a dose-dependent decrease in Nrf2 activity using ARE-luciferase reporters [33,37]. Also Cavin *et al.* demonstrated, using an electrophoretic mobility shift assay, that OTA exposure decreases Nrf2 DNA binding in rat hepatocytes [33]. Interestingly, hepatocytes pre-treated with the coffee diterpenes combination of Cafestol and Kahweol, which is a strong inducer of Nrf2 [38], maintained a robust Nrf2 response in the presence of OTA. However, Nrf2 induction was significantly decreased when OTA was co-incubated with the diterpenes. Thus OTA does not interfere with an ongoing Nrf2 response, but does block the initiation of the response. Although, these studies demonstrate that OTA interferes with Nrf2 DNA binding, they do not exclude the possibility of an inhibition of Nrf2 mobilisation.

There is also a growing weight of evidence showing epigenomic effects of OTA. OTA has been shown both to increase histone deacetylase 3 (HDAC3) expression [32] and histone acetyltransferases (HAT) inhibition [10]. It has also been shown that genes governing histone regulation are induced by OTA, including Jumonji domain containing 6 (Jmjd6), which demethylates histones H3 and H4 [5]. Also a number of histone regulating genes are decreased by OTA, including death-associated protein kinase 3 (Dapk3) H3 and H4 kinase, Zinc finger, MYM-type 3 (Zmym3) a proposed member of the histone deacetylase-containing multiprotein complexes and TAF5-like RNA polymerase II, p300/CBP-associated factor (PCAF)-associated factor (Taf5l) [5]. Taf5l is a member of the PCAF complex which promotes histone acetylation [5]. Thus OTA perturbs gene regulation possibly through promotion of histone hypo-acetylation rendering DNA less accessible for binding of transcriptional machinery. Such an effect would negatively affect transcription factor DNA binding, including Nrf2/ARE binding, contributing to a decrease in Nrf2-dependent gene transcription.

In the last number of years, it has been demonstrated that microRNAs (miRNAs) are important post transcriptional regulators and some have also been demonstrated to affect Nrf2 and its downstream targets. OTA has also been shown to induce several miRNAs, with an especially high induction of miR-132 in LLC-PK1 cells [39]. This was associated with a decrease of Nrf2 mRNA [39]. Additionally, it was shown that silencing miR-132 with an antigomir, prevented the decrease of Nrf2 mRNA upon OTA exposure [39]. These results suggest an implication of miRNAs, especially miR-132, in the regulation of the Nrf2 gene itself. Long term OTA induction of miR-132 would lead to a depletion of Nrf2 protein pools which would finally result in a complete Nrf2 block.

**Figure 2:**
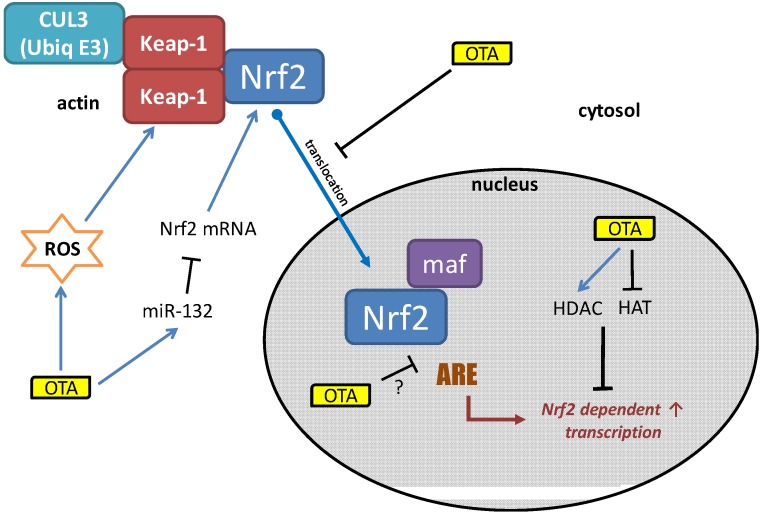
Elucidated mechanisms of OTA induced Nrf2 inhibition.

OTA can inhibit the activation of the Nrf2 pathway at multiple points, as summarized in Figure 2. One of the key events is the inhibition of Nrf2 mobilization and thus translocation to the nucleus. While, the mechanism for this has not yet been elucidated, it has been shown that OTA binds strongly to actin filaments [40]. This would likely interfere with cytoskeletal dynamics. For example we have shown that OTA strongly induced advillin, a member of the gelsolin superfamily which plays a role in actin remodeling [5]. Since Keap-1 is an actin-bound protein, it is possible that OTA prevents the liberation of Nrf2 from the Keap-1 complex. Alternatively, OTA may itself bind Nrf2 and hold it in the cytosol, mimicking Keap-1’s function. Such an action would prevent Nrf2 nuclear translocation and thus binding to the ARE DNA motif regardless of the amount of oxidative equivalents present. Additionally, OTA has epigenetic effects and inhibits HAT activity while inducing HDACs. This would decrease global transcription, including Nrf2-dependent genes. Finally, OTA has been shown to induce miR-132 which targets Nrf2 mRNA and would thus lead to a decrease in Nrf2 protein pools.

In conclusion, OTA-induced inhibition of Nrf2 activation and Nrf2 gene transcription together with OTA-induced Nrf2 protein depletion would render the cell defenseless to physiological and compound-induced oxidative stress. This, coupled with the facts that OTA itself induces oxidative stress, and that Nrf2 induction prior to OTA exposure prevents OTA-induced cell death, provides strong evidence that Nrf2 inhibition is the primary mechanism of OTA’s toxicity. Nrf2 inhibition and subsequent increase in ROS would lead to lipid peroxidation, proteotoxic stress and oxidative DNA damage. Other protective pathways would inevitably be activated, such as p53, to prevent the cell replicating by initiating cell cycle blockade and promoting apoptosis. However, over a chronic time course such a situation will create a selective pressure leading to escape from p53 activity and thus promotion of a cancerous phenotype. Additionally, Nrf2 inhibition alone may select for cells with somatic mutations in Nrf2 or Keap-1, leading to a constitutive activation of the pathway. A cell without p53 control and a constitutively active Nrf2 system would have a massive survival advantage and would also be very resistant to chemotherapeutics. Indeed, utilizing the high Nrf2 expression in tumor cells is now being considered as a method for tumor specific targeting of toxic payloads [41]. In conclusion, there is compelling evidence that OTA’s inhibition of Nrf2 is the mechanism for both its nephrotoxicity and carcinogenicity.

## References

[1] Clark H.A., Snedeker S.M. (2006). Ochratoxin A: Its cancer risk and potential for exposure. J. Toxicol. Environ. Health B Crit. Rev..

[2] Scientific Cooperation (SCOOP) Task Reports Reports on tasks for scientific cooperation: Assessment of dietary intake of ochratoxin a by the population of eu member states. http://ec.europa.eu/food/fs/scoop/3.2.7_en.pdf.

[3] Zlender V., Breljak D., Ljubojevic M., Flajs D., Balen D., Brzica H., Domijan A.M., Peraica M., Fuchs R., Anzai N. (2009). Low doses of ochratoxin a upregulate the protein expression of organic anion transporters oat1, oat2, oat3 and oat5 in rat kidney cortex. Toxicol. Appl. Pharmacol..

[4] Anzai N., Jutabha P., Endou H. (2010). Molecular mechanism of ochratoxin a transport in the kidney. Toxins.

[5] Jennings P., Weiland C., Limonciel A., Bloch K.M., Radford R., Aschauer L., McMorrow T., Wilmes A., Pfaller W., Ahr H.J. (2012). Transcriptomic alterations induced by ochratoxin A in rat and human renal proximal tubular *in vitro* models and comparison to a rat *in vivo* model. Arch. Toxicol..

[6] Boorman G.A., McDonald M.R., Imoto S., Persing R. (1992). Renal lesions induced by ochratoxin a exposure in the f344 rat. Toxicol. Pathol..

[7] Boorman G.A. (1989). Toxicology and carcinogenesis studies of ochratoxin a in f344/n rats (gavage studies). Natl. Toxicol. Progr. Tech. Rep. Ser..

[8] Pfohl-Leszkowicz A., Manderville R.A. (2012). An update on direct genotoxicity as a molecular mechanism of ochratoxin a carcinogenicity. Chem. Res. Toxicol..

[9] Rached E., Pfeiffer E., Dekant W., Mally A. (2006). Ochratoxin A: Apoptosis and aberrant exit from mitosis due to perturbation of microtubule dynamics?. Toxicol. Sci..

[10] Czakai K., Muller K., Mosesso P., Pepe G., Schulze M., Gohla A., Patnaik D., Dekant W., Higgins J.M., Mally A. (2011). Perturbation of mitosis through inhibition of histone acetyltransferases: The key to ochratoxin a toxicity and carcinogenicity?. Toxicol. Sci..

[11] Jennings P., Limonciel A., Felice L., Leonard M.O. (2013). An overview of transcriptional regulation in response to toxicological insult. Arch. Toxicol..

[12] Itoh K., Chiba T., Takahashi S., Ishii T., Igarashi K., Katoh Y., Oyake T., Hayashi N., Satoh K., Hatayama I. (1997). An Nrf2/small maf heterodimer mediates the induction of phase ii detoxifying enzyme genes through antioxidant response elements. Biochem. Biophys. Res. Commun..

[13] Baird L., Dinkova-Kostova A.T. (2011). The cytoprotective role of the keap1-Nrf2 pathway. Arch. Toxicol..

[14] Yates M.S., Tran Q.T., Dolan P.M., Osburn W.O., Shin S., McCulloch C.C., Silkworth J.B., Taguchi K., Yamamoto M., Williams C.R. (2009). Genetic *versus* chemoprotective activation of Nrf2 signaling: Overlapping yet distinct gene expression profiles between keap1 knockout and triterpenoid-treated mice. Carcinogenesis.

[15] Okawa H., Motohashi H., Kobayashi A., Aburatani H., Kensler T.W., Yamamoto M. (2006). Hepatocyte-specific deletion of the keap1 gene activates Nrf2 and confers potent resistance against acute drug toxicity. Biochem. Biophys. Res. Commun..

[16] Reichard J.F., Motz G.T., Puga A. (2007). Heme oxygenase-1 induction by Nrf2 requires inactivation of the transcriptional repressor bach1. Nucleic Acids Res..

[17] Yueh M.F., Tukey R.H. (2007). Nrf2-keap1 signaling pathway regulates human ugt1a1 expression *in vitro* and in transgenic ugt1 mice. J. Biol. Chem..

[18] Harvey C.J., Thimmulappa R.K., Singh A., Blake D.J., Ling G., Wakabayashi N., Fujii J., Myers A., Biswal S. (2009). Nrf2-regulated glutathione recycling independent of biosynthesis is critical for cell survival during oxidative stress. Free Radic. Biol. Med..

[19] Jung K.A., Choi B.H., Nam C.W., Song M., Kim S.T., Lee J.Y., Kwak M.K. (2013). Identification of aldo-keto reductases as Nrf2-target marker genes in human cells. Toxicol. Lett..

[20] Limonciel A., Wilmes A., Aschauer L., Radford R., Bloch K.M., McMorrow T., Pfaller W., van Delft J.H., Slattery C., Ryan M.P. (2012). Oxidative stress induced by potassium bromate exposure results in altered tight junction protein expression in renal proximal tubule cells. Arch. Toxicol..

[21] Wilmes A., Crean D., Aydin S., Pfaller W., Jennings P., Leonard M.O. (2011). Identification and dissection of the Nrf2 mediated oxidative stress pathway in human renal proximal tubule toxicity. Toxicol. in Vitro.

[22] Wilmes A., Limonciel A., Aschauer L., Moenks K., Bielow C., Leonard M.O., Hamon J., Carpi D., Ruzek S., Handler A. (2013). Application of integrated transcriptomic, proteomic and metabolomic profiling for the delineation of mechanisms of drug induced cell stress. J. Proteomics.

[23] Forti E., Bulgheroni A., Cetin Y., Hartung T., Jennings P., Pfaller W., Prieto P. (2010). Characterisation of cadmium chloride induced molecular and functional alterations in airway epithelial cells. Cell. Phys. Biochem..

[24] Forti E., Salovaara S., Cetin Y., Bulgheroni A., Tessadri R., Jennings P., Pfaller W., Prieto P. (2011). *In vitro* evaluation of the toxicity induced by nickel soluble and particulate forms in human airway epithelial cells. Toxicol. in Vitro.

[25] Crean D., Felice L., Taylor C.T., Rabb H., Jennings P., Leonard M.O. (2012). Glucose reintroduction triggers the activation of nrf2 during experimental ischemia reperfusion. Mol. Cell. Biochem..

[26] Liu F., Ichihara S., Valentine W.M., Itoh K., Yamamoto M., Sheik Mohideen S., Kitoh J., Ichihara G. (2010). Increased susceptibility of nrf2-null mice to 1-bromopropane-induced hepatotoxicity. Toxicol. Sci..

[27] Copple I.M., Goldring C.E., Kitteringham N.R., Park B.K. (2008). The nrf2-keap1 defence pathway: Role in protection against drug-induced toxicity. Toxicology.

[28] Aleksunes L.M., Goedken M.J., Rockwell C.E., Thomale J., Manautou J.E., Klaassen C.D. (2010). Transcriptional regulation of renal cytoprotective genes by nrf2 and its potential use as a therapeutic target to mitigate cisplatin-induced nephrotoxicity. J. Pharmacol. Exp. Ther..

[29] Enomoto A., Itoh K., Nagayoshi E., Haruta J., Kimura T., O’Connor T., Harada T., Yamamoto M. (2001). High sensitivity of nrf2 knockout mice to acetaminophen hepatotoxicity associated with decreased expression of are-regulated drug metabolizing enzymes and antioxidant genes. Toxicol. Sci..

[30] Sorrenti V., Di Giacomo C., Acquaviva R., Barbagallo I., Bognanno M., Galvano F. (2013). Toxicity of ochratoxin a and its modulation by antioxidants: A review. Toxins.

[31] Schaaf G.J., Nijmeijer S.M., Maas R.F., Roestenberg P., de Groene E.M., Fink-Gremmels J. (2002). The role of oxidative stress in the ochratoxin a-mediated toxicity in proximal tubular cells. Biochim. Biophys. Acta.

[32] Marin-Kuan M., Nestler S., Verguet C., Bezencon C., Piguet D., Mansourian R., Holzwarth J., Grigorov M., Delatour T., Mantle P. (2006). A toxicogenomics approach to identify new plausible epigenetic mechanisms of ochratoxin a carcinogenicity in rat. Toxicol. Sci..

[33] Cavin C., Delatour T., Marin-Kuan M., Holzhauser D., Higgins L., Bezencon C., Guignard G., Junod S., Richoz-Payot J., Gremaud E. (2007). Reduction in antioxidant defenses may contribute to ochratoxin a toxicity and carcinogenicity. Toxicol. Sci..

[34] Kumar R., Ansari K.M., Chaudhari B.P., Dhawan A., Dwivedi P.D., Jain S.K., Das M. (2012). Topical application of ochratoxin a causes DNA damage and tumor initiation in mouse skin. PloS ONE.

[35] Ramyaa P., Krishnaswamy R., Padma V.V. (2013). Quercetin modulates ota-induced oxidative stress and redox signalling in hepg2 cells—Up regulation of nrf2 expression and down regulation of nf-kappab and cox-2. Biochim. Biophys. Acta.

[36] Boesch-Saadatmandi C., Wagner A.E., Graeser A.C., Hundhausen C., Wolffram S., Rimbach G. (2009). Ochratoxin a impairs Nrf2-dependent gene expression in porcine kidney tubulus cells. J. Anim. Physiol. Anim. Nutr..

[37] Boesch-Saadatmandi C., Loboda A., Jozkowicz A., Huebbe P., Blank R., Wolffram S., Dulak J., Rimbach G. (2008). Effect of ochratoxin a on redox-regulated transcription factors, antioxidant enzymes and glutathione-s-transferase in cultured kidney tubulus cells. Food Chem. Toxicol..

[38] Cavin C., Holzhaeuser D., Scharf G., Constable A., Huber W.W., Schilter B. (2002). Cafestol and kahweol, two coffee specific diterpenes with anticarcinogenic activity. Food Chem. Toxicol..

[39] Stachurska A., Ciesla M., Kozakowska M., Wolffram S., Boesch-Saadatmandi C., Rimbach G., Jozkowicz A., Dulak J., Loboda A. (2013). Cross-talk between micrornas, nuclear factor e2-related factor 2, and heme oxygenase-1 in ochratoxin a-induced toxic effects in renal proximal tubular epithelial cells. Mol. Nutr. Food Res..

[40] Dopp E., Muller J., Hahnel C., Schiffmann D. (1999). Induction of genotoxic effects and modulation of the intracellular calcium level in syrian hamster embryo (she) fibroblasts caused by ochratoxin A. Food Chem. Toxicol..

[41] Kansanen E., Kuosmanen S.M., Leinonen H., Levonen A.L. (2013). The Keap1-Nrf2 pathway: Mechanisms of activation and dysregulation in cancer. Redox Biol..

